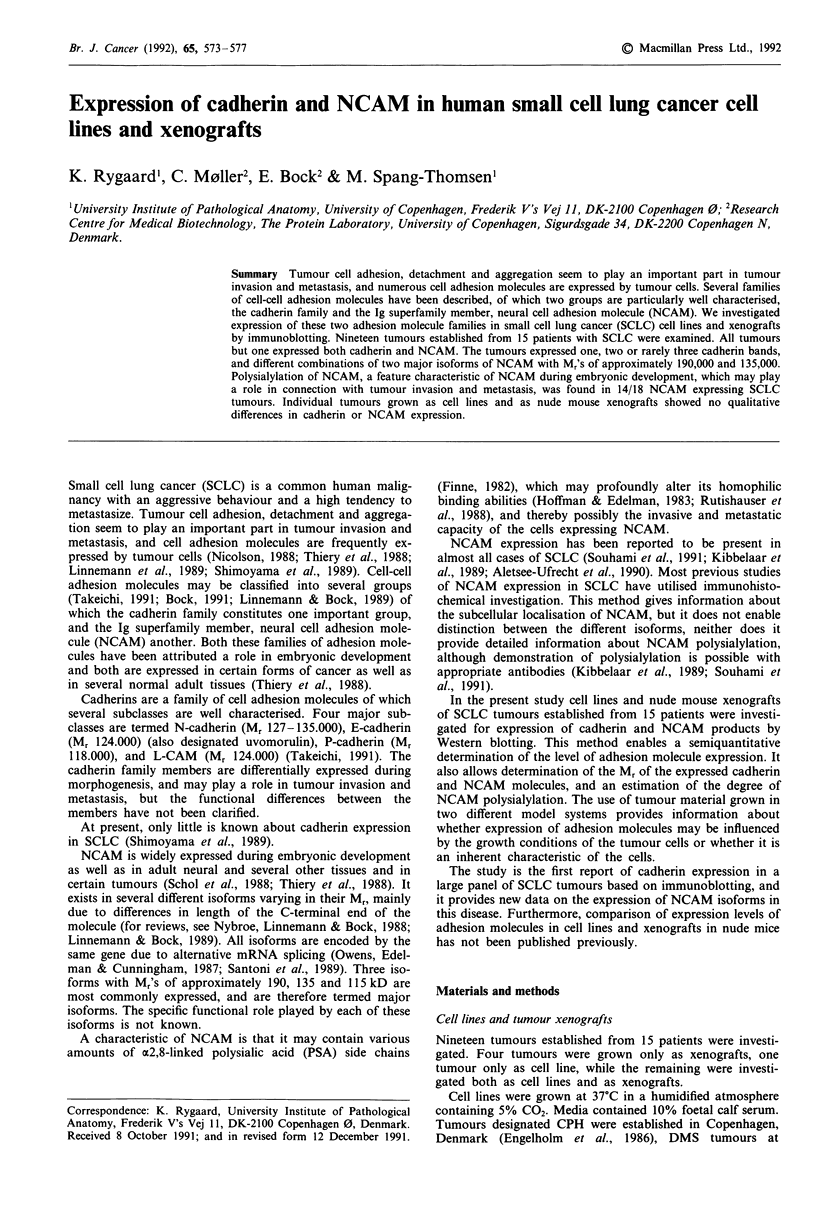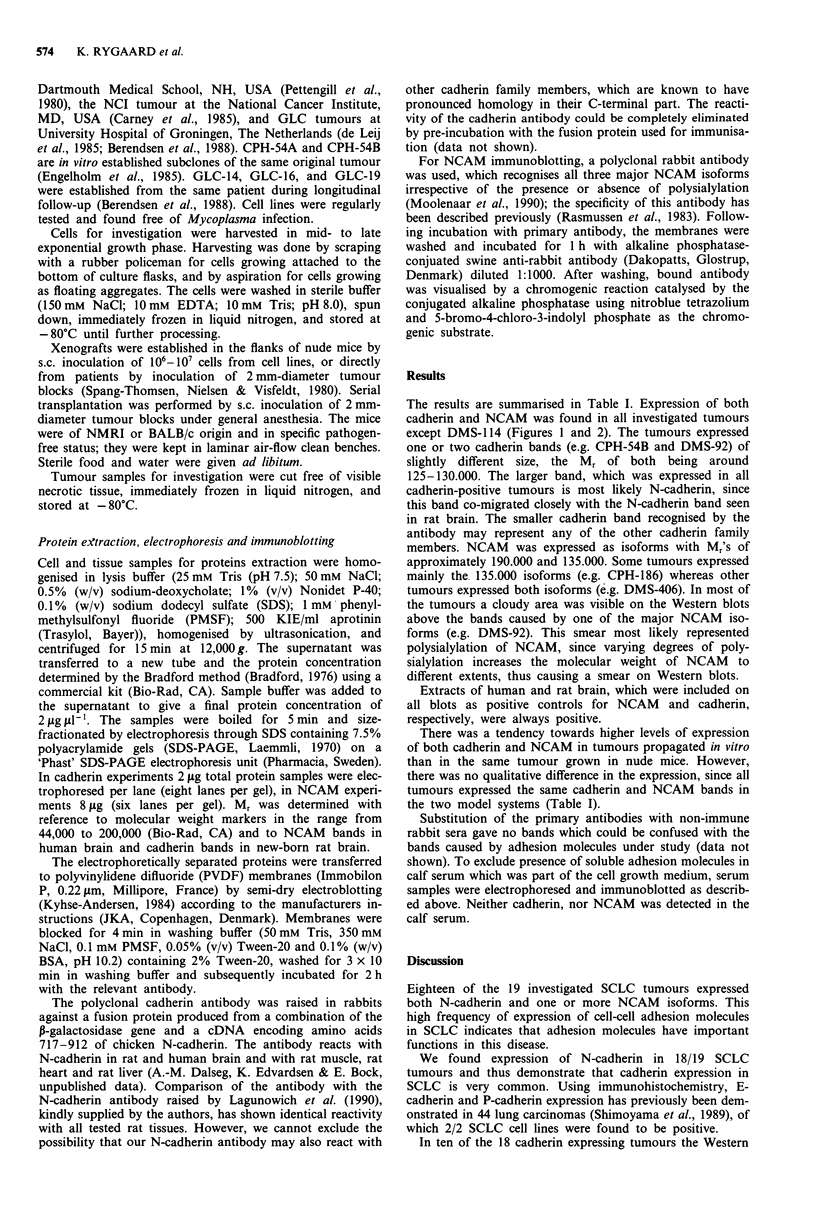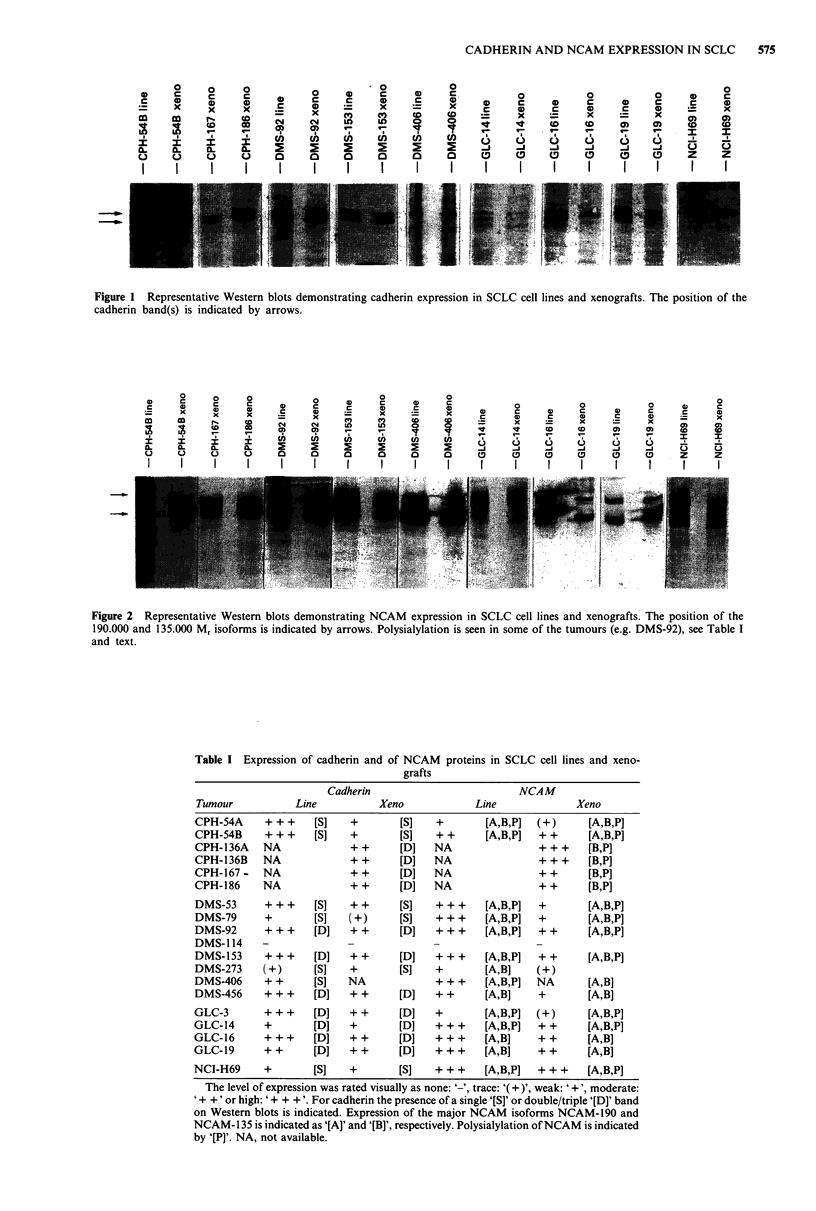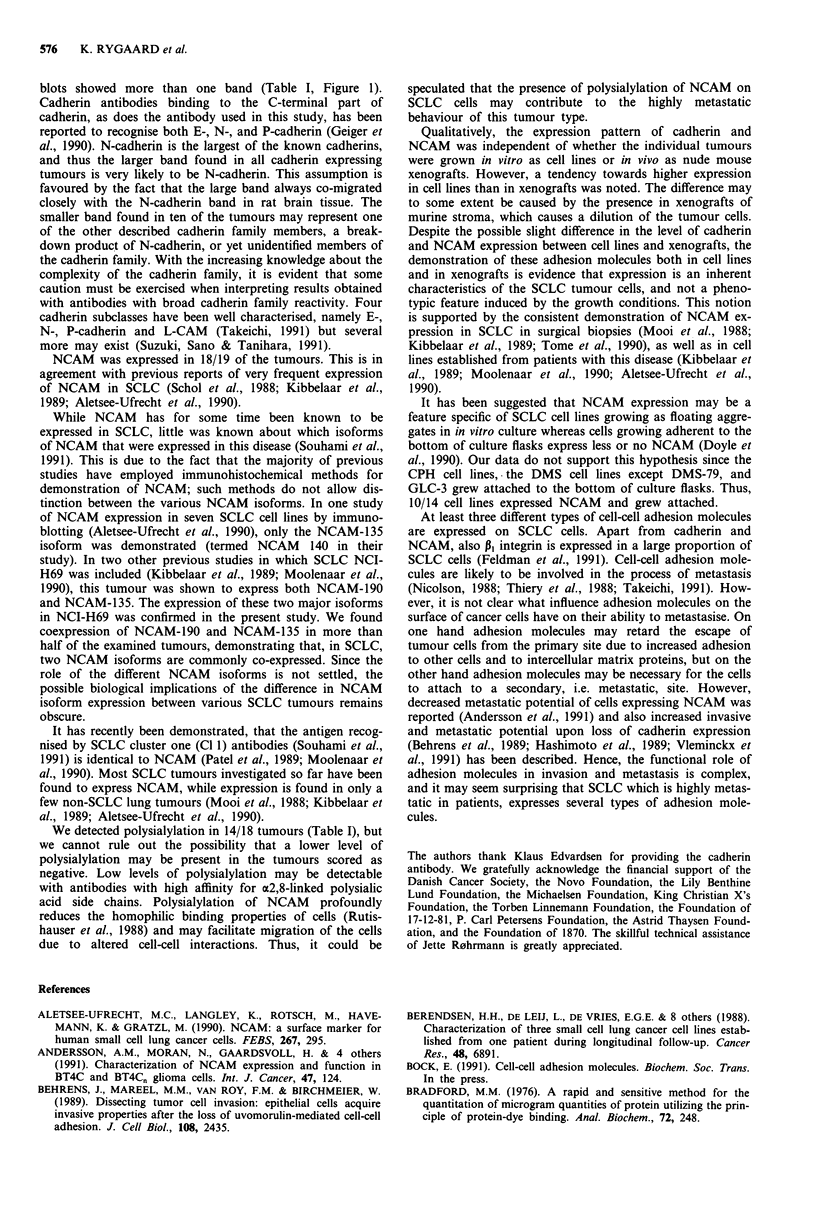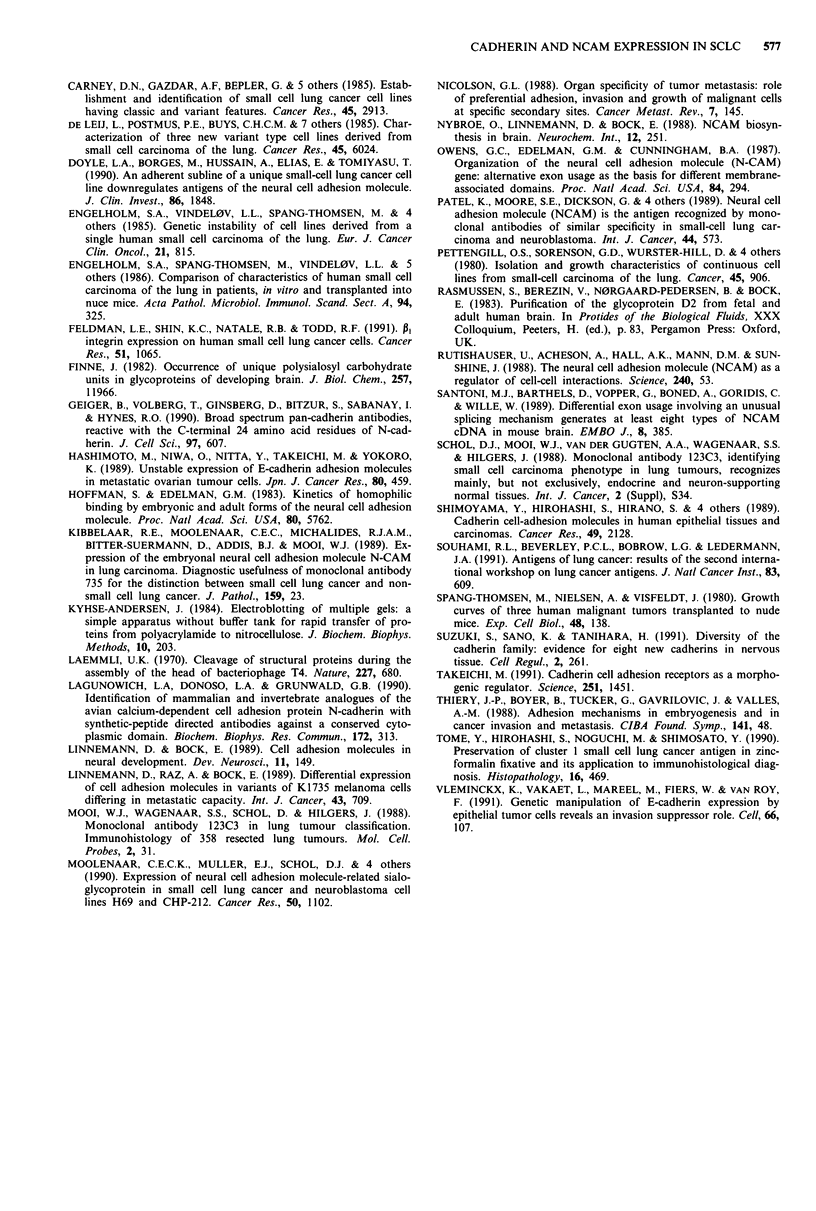# Expression of cadherin and NCAM in human small cell lung cancer cell lines and xenografts.

**DOI:** 10.1038/bjc.1992.116

**Published:** 1992-04

**Authors:** K. Rygaard, C. Møller, E. Bock, M. Spang-Thomsen

**Affiliations:** University Institute of Pathological Anatomy, University of Copenhagen, Denmark.

## Abstract

**Images:**


Br. J. Cancer (1992), **65**, 573–577 © Macmillan Press Ltd., 1992

### Expression of cadherin and NCAM in human small cell lung cancer cell lines and xenografts

K. Rygaard[1], C. Møller[2], E. Bock[2] & M. Spang-Thomsen[1]

[1]*University Institute of Pathological Anatomy, University of Copenhagen, Frederik V's Vej 11, DK-2100 Copenhagen Ø;* [2]*Research Centre for Medical Biotechnology, The Protein Laboratory, University of Copenhagen, Sigurdsgade 34, DK-2200 Copenhagen N, Denmark.*

**Summary** Tumour cell adhesion, detachment and aggregation seem to play an important part in tumour invasion and metastasis, and numerous cell adhesion molecules are expressed by tumour cells. Several families of cell-cell adhesion molecules have been described, of which two groups are particularly well characterised, the cadherin family and the Ig superfamily member, neural cell adhesion molecule (NCAM). We investigated expression of these two adhesion molecule families in small cell lung cancer (SCLC) cell lines and xenografts by immunoblotting. Nineteen tumours established from 15 patients with SCLC were examined. All tumours but one expressed both cadherin and NCAM. The tumours expressed one, two or rarely three cadherin bands, and different combinations of two major isoforms of NCAM with $M_r$'s of approximately 190,000 and 135,000. Polysialylation of NCAM, a feature characteristic of NCAM during embryonic development, which may play a role in connection with tumour invasion and metastasis, was found in 14/18 NCAM expressing SCLC tumours. Individual tumours grown as cell lines and as nude mouse xenografts showed no qualitative differences in cadherin or NCAM expression.

Small cell lung cancer (SCLC) is a common human malignancy with an aggressive behaviour and a high tendency to metastasize. Tumour cell adhesion, detachment and aggregation seem to play an important part in tumour invasion and metastasis, and cell adhesion molecules are frequently expressed by tumour cells (Nicolson, 1988; Thiery *et al.*, 1988; Linnemann *et al.*, 1989; Shimoyama *et al.*, 1989). Cell-cell adhesion molecules may be classified into several groups (Takeichi, 1991; Bock, 1991; Linnemann & Bock, 1989) of which the cadherin family constitutes one important group, and the Ig superfamily member, neural cell adhesion molecule (NCAM) another. Both these families of adhesion molecules have been attributed a role in embryonic development and both are expressed in certain forms of cancer as well as in several normal adult tissues (Thiery *et al.*, 1988).

Cadherins are a family of cell adhesion molecules of which several subclasses are well characterised. Four major subclasses are termed N-cadherin ($M_r$ 127–135.000), E-cadherin ($M_r$ 124.000) (also designated uvomorulin), P-cadherin ($M_r$ 118.000), and L-CAM ($M_r$ 124.000) (Takeichi, 1991). The cadherin family members are differentially expressed during morphogenesis, and may play a role in tumour invasion and metastasis, but the functional differences between the members have not been clarified.

At present, only little is known about cadherin expression in SCLC (Shimoyama *et al.*, 1989).

NCAM is widely expressed during embryonic development as well as in adult neural and several other tissues and in certain tumours (Schol *et al.*, 1988; Thiery *et al.*, 1988). It exists in several different isoforms varying in their $M_r$, mainly due to differences in length of the C-terminal end of the molecule (for reviews, see Nybroe, Linnemann & Bock, 1988; Linnemann & Bock, 1989). All isoforms are encoded by the same gene due to alternative mRNA splicing (Owens, Edelman & Cunningham, 1987; Santoni *et al.*, 1989). Three isoforms with $M_r$'s of approximately 190, 135 and 115 kD are most commonly expressed, and are therefore termed major isoforms. The specific functional role played by each of these isoforms is not known.

A characteristic of NCAM is that it may contain various amounts of α2,8-linked polysialic acid (PSA) side chains (Finne, 1982), which may profoundly alter its homophilic binding abilities (Hoffman & Edelman, 1983; Rutishauser *et al.*, 1988), and thereby possibly the invasive and metastatic capacity of the cells expressing NCAM.

NCAM expression has been reported to be present in almost all cases of SCLC (Souhami *et al.*, 1991; Kibbelaar *et al.*, 1989; Aletsee-Ufrecht *et al.*, 1990). Most previous studies of NCAM expression in SCLC have utilised immunohistochemical investigation. This method gives information about the subcellular localisation of NCAM, but it does not enable distinction between the different isoforms, neither does it provide detailed information about NCAM polysialylation, although demonstration of polysialylation is possible with appropriate antibodies (Kibbelaar *et al.*, 1989; Souhami *et al.*, 1991).

In the present study cell lines and nude mouse xenografts of SCLC tumours established from 15 patients were investigated for expression of cadherin and NCAM products by Western blotting. This method enables a semiquantitative determination of the level of adhesion molecule expression. It also allows determination of the $M_r$ of the expressed cadherin and NCAM molecules, and an estimation of the degree of NCAM polysialylation. The use of tumour material grown in two different model systems provides information about whether expression of adhesion molecules may be influenced by the growth conditions of the tumour cells or whether it is an inherent characteristic of the cells.

The study is the first report of cadherin expression in a large panel of SCLC tumours based on immunoblotting, and it provides new data on the expression of NCAM isoforms in this disease. Furthermore, comparison of expression levels of adhesion molecules in cell lines and xenografts in nude mice has not been published previously.

### Materials and methods

### *Cell lines and tumour xenografts*

Nineteen tumours established from 15 patients were investigated. Four tumours were grown only as xenografts, one tumour only as cell line, while the remaining were investigated both as cell lines and as xenografts.

Cell lines were grown at 37°C in a humidified atmosphere containing 5% $CO_2$. Media contained 10% foetal calf serum. Tumours designated CPH were established in Copenhagen, Denmark (Engelholm *et al.*, 1986), DMS tumours at

Correspondence: K. Rygaard, University Institute of Pathological Anatomy, Frederik V's Vej 11, DK-2100 Copenhagen Ø, Denmark.
Received 8 October 1991; and in revised form 12 December 1991.

Dartmouth Medical School, NH, USA (Pettengill et al., 1980), the NCI tumour at the National Cancer Institute, MD, USA (Carney et al., 1985), and GLC tumours at University Hospital of Groningen, The Netherlands (de Leij et al., 1985; Berendsen et al., 1988). CPH-54A and CPH-54B are *in vitro* established subclones of the same original tumour (Engelholm et al., 1985). GLC-14, GLC-16, and GLC-19 were established from the same patient during longitudinal follow-up (Berendsen et al., 1988). Cell lines were regularly tested and found free of *Mycoplasma* infection.

Cells for investigation were harvested in mid- to late exponential growth phase. Harvesting was done by scraping with a rubber policeman for cells growing attached to the bottom of culture flasks, and by aspiration for cells growing as floating aggregates. The cells were washed in sterile buffer (150 mM NaCl; 10 mM EDTA; 10 mM Tris; pH 8.0), spun down, immediately frozen in liquid nitrogen, and stored at −80°C until further processing.

Xenografts were established in the flanks of nude mice by s.c. inoculation of $10^6-10^7$ cells from cell lines, or directly from patients by inoculation of 2 mm-diameter tumour blocks (Spang-Thomsen, Nielsen & Visfeldt, 1980). Serial transplantation was performed by s.c. inoculation of 2 mm-diameter tumour blocks under general anesthesia. The mice were of NMRI or BALB/c origin and in specific pathogen-free status; they were kept in laminar air-flow clean benches. Sterile food and water were given *ad libitum*.

Tumour samples for investigation were cut free of visible necrotic tissue, immediately frozen in liquid nitrogen, and stored at −80°C.

*Protein extraction, electrophoresis and immunoblotting*

Cell and tissue samples for proteins extraction were homogenised in lysis buffer (25 mM Tris (pH 7.5); 50 mM NaCl; 0.5% (w/v) sodium-deoxycholate; 1% (v/v) Nonidet P-40; 0.1% (w/v) sodium dodecyl sulfate (SDS); 1 mM phenylmethylsulfonyl fluoride (PMSF); 500 KIE/ml aprotinin (Trasylol, Bayer)), homogenised by ultrasonication, and centrifuged for 15 min at 12,000 *g*. The supernatant was transferred to a new tube and the protein concentration determined by the Bradford method (Bradford, 1976) using a commercial kit (Bio-Rad, CA). Sample buffer was added to the supernatant to give a final protein concentration of $2\ \mu g\ \mu l^{-1}$. The samples were boiled for 5 min and size-fractionated by electrophoresis through SDS containing 7.5% polyacrylamide gels (SDS-PAGE, Laemmli, 1970) on a 'Phast' SDS-PAGE electrophoresis unit (Pharmacia, Sweden). In cadherin experiments 2 µg total protein samples were electrophoresed per lane (eight lanes per gel), in NCAM experiments 8 µg (six lanes per gel). $M_r$ was determined with reference to molecular weight markers in the range from 44,000 to 200,000 (Bio-Rad, CA) and to NCAM bands in human brain and cadherin bands in new-born rat brain.

The electrophoretically separated proteins were transferred to polyvinylidene difluoride (PVDF) membranes (Immobilon P, 0.22 µm, Millipore, France) by semi-dry electroblotting (Kyhse-Andersen, 1984) according to the manufacturers instructions (JKA, Copenhagen, Denmark). Membranes were blocked for 4 min in washing buffer (50 mM Tris, 350 mM NaCl, 0.1 mM PMSF, 0.05% (v/v) Tween-20 and 0.1% (w/v) BSA, pH 10.2) containing 2% Tween-20, washed for 3 × 10 min in washing buffer and subsequently incubated for 2 h with the relevant antibody.

The polyclonal cadherin antibody was raised in rabbits against a fusion protein produced from a combination of the β-galactosidase gene and a cDNA encoding amino acids 717–912 of chicken N-cadherin. The antibody reacts with N-cadherin in rat and human brain and with rat muscle, rat heart and rat liver (A.-M. Dalseg, K. Edvardsen & E. Bock, unpublished data). Comparison of the antibody with the N-cadherin antibody raised by Lagunowich et al. (1990), kindly supplied by the authors, has shown identical reactivity with all tested rat tissues. However, we cannot exclude the possibility that our N-cadherin antibody may also react with other cadherin family members, which are known to have pronounced homology in their C-terminal part. The reactivity of the cadherin antibody could be completely eliminated by pre-incubation with the fusion protein used for immunisation (data not shown).

For NCAM immunoblotting, a polyclonal rabbit antibody was used, which recognises all three major NCAM isoforms irrespective of the presence or absence of polysialylation (Moolenaar et al., 1990); the specificity of this antibody has been described previously (Rasmussen et al., 1983). Following incubation with primary antibody, the membranes were washed and incubated for 1 h with alkaline phosphatase-conjuated swine anti-rabbit antibody (Dakopatts, Glostrup, Denmark) diluted 1:1000. After washing, bound antibody was visualised by a chromogenic reaction catalysed by the conjugated alkaline phosphatase using nitroblue tetrazolium and 5-bromo-4-chloro-3-indolyl phosphate as the chromogenic substrate.

### Results

The results are summarised in Table I. Expression of both cadherin and NCAM was found in all investigated tumours except DMS-114 (Figures 1 and 2). The tumours expressed one or two cadherin bands (e.g. CPH-54B and DMS-92) of slightly different size, the $M_r$ of both being around 125–130.000. The larger band, which was expressed in all cadherin-positive tumours is most likely N-cadherin, since this band co-migrated closely with the N-cadherin band seen in rat brain. The smaller cadherin band recognised by the antibody may represent any of the other cadherin family members. NCAM was expressed as isoforms with $M_r$'s of approximately 190.000 and 135.000. Some tumours expressed mainly the 135.000 isoforms (e.g. CPH-186) whereas other tumours expressed both isoforms (e.g. DMS-406). In most of the tumours a cloudy area was visible on the Western blots above the bands caused by one of the major NCAM isoforms (e.g. DMS-92). This smear most likely represented polysialylation of NCAM, since varying degrees of polysialylation increases the molecular weight of NCAM to different extents, thus causing a smear on Western blots.

Extracts of human and rat brain, which were included on all blots as positive controls for NCAM and cadherin, respectively, were always positive.

There was a tendency towards higher levels of expression of both cadherin and NCAM in tumours propagated *in vitro* than in the same tumour grown in nude mice. However, there was no qualitative difference in the expression, since all tumours expressed the same cadherin and NCAM bands in the two model systems (Table I).

Substitution of the primary antibodies with non-immune rabbit sera gave no bands which could be confused with the bands caused by adhesion molecules under study (data not shown). To exclude presence of soluble adhesion molecules in calf serum which was part of the cell growth medium, serum samples were electrophoresed and immunoblotted as described above. Neither cadherin, nor NCAM was detected in the calf serum.

### Discussion

Eighteen of the 19 investigated SCLC tumours expressed both N-cadherin and one or more NCAM isoforms. This high frequency of expression of cell-cell adhesion molecules in SCLC indicates that adhesion molecules have important functions in this disease.

We found expression of N-cadherin in 18/19 SCLC tumours and thus demonstrate that cadherin expression in SCLC is very common. Using immunohistochemistry, E-cadherin and P-cadherin expression has previously been demonstrated in 44 lung carcinomas (Shimoyama et al., 1989), of which 2/2 SCLC cell lines were found to be positive.

In ten of the 18 cadherin expressing tumours the Western

**Figure 1** Representative Western blots demonstrating cadherin expression in SCLC cell lines and xenografts. The position of the cadherin band(s) is indicated by arrows.

**Figure 2** Representative Western blots demonstrating NCAM expression in SCLC cell lines and xenografts. The position of the 190.000 and 135.000 $M_r$ isoforms is indicated by arrows. Polysialylation is seen in some of the tumours (e.g. DMS-92), see Table I and text.

**Table I** Expression of cadherin and of NCAM proteins in SCLC cell lines and xenografts

| | *Cadherin* | | | | *NCAM* | | | |
|---|---|---|---|---|---|---|---|---|
| *Tumour* | *Line* | | *Xeno* | | *Line* | | *Xeno* | |
| CPH-54A | + + + | [S] | + | [S] | + | [A,B,P] | (+) | [A,B,P] |
| CPH-54B | + + + | [S] | + | [S] | + + | [A,B,P] | + + | [A,B,P] |
| CPH-136A | NA | | + + | [D] | NA | | + + + | [B,P] |
| CPH-136B | NA | | + + | [D] | NA | | + + + | [B,P] |
| CPH-167 | NA | | + + | [D] | NA | | + + | [B,P] |
| CPH-186 | NA | | + + | [D] | NA | | + + | [B,P] |
| DMS-53 | + + + | [S] | + + | [S] | + + + | [A,B,P] | + | [A,B,P] |
| DMS-79 | + | [S] | (+) | [S] | + + + | [A,B,P] | + | [A,B,P] |
| DMS-92 | + + + | [D] | + + | [D] | + + + | [A,B,P] | + + | [A,B,P] |
| DMS-114 | – | | – | | – | | – | |
| DMS-153 | + + + | [D] | + + | [D] | + + + | [A,B,P] | + + | [A,B,P] |
| DMS-273 | (+) | [S] | + | [S] | + | [A,B] | (+) | |
| DMS-406 | + + | [S] | NA | | + + + | [A,B,P] | NA | [A,B] |
| DMS-456 | + + + | [D] | + + | [D] | + + | [A,B] | + | [A,B] |
| GLC-3 | + + + | [D] | + + | [D] | + | [A,B,P] | (+) | [A,B,P] |
| GLC-14 | + | [D] | + | [D] | + + + | [A,B,P] | + + | [A,B,P] |
| GLC-16 | + + + | [D] | + + | [D] | + + + | [A,B] | + + | [A,B] |
| GLC-19 | + + | [D] | + + | [D] | + + + | [A,B] | + + | [A,B] |
| NCI-H69 | + | [S] | + | [S] | + + + | [A,B,P] | + + + | [A,B,P] |

The level of expression was rated visually as none: '–', trace: '(+)', weak: '+', moderate: '+ +' or high: '+ + +'. For cadherin the presence of a single '[S]' or double/triple '[D]' band on Western blots is indicated. Expression of the major NCAM isoforms NCAM-190 and NCAM-135 is indicated as '[A]' and '[B]', respectively. Polysialylation of NCAM is indicated by '[P]'. NA, not available.

blots showed more than one band (Table I, Figure 1). Cadherin antibodies binding to the C-terminal part of cadherin, as does the antibody used in this study, has been reported to recognise both E-, N-, and P-cadherin (Geiger *et al.*, 1990). N-cadherin is the largest of the known cadherins, and thus the larger band found in all cadherin expressing tumours is very likely to be N-cadherin. This assumption is favoured by the fact that the large band always co-migrated closely with the N-cadherin band in rat brain tissue. The smaller band found in ten of the tumours may represent one of the other described cadherin family members, a breakdown product of N-cadherin, or yet unidentified members of the cadherin family. With the increasing knowledge about the complexity of the cadherin family, it is evident that some caution must be exercised when interpreting results obtained with antibodies with broad cadherin family reactivity. Four cadherin subclasses have been well characterised, namely E-, N-, P-cadherin and L-CAM (Takeichi, 1991) but several more may exist (Suzuki, Sano & Tanihara, 1991).

NCAM was expressed in 18/19 of the tumours. This is in agreement with previous reports of very frequent expression of NCAM in SCLC (Schol *et al.*, 1988; Kibbelaar *et al.*, 1989; Aletsee-Ufrecht *et al.*, 1990).

While NCAM has for some time been known to be expressed in SCLC, little was known about which isoforms of NCAM that were expressed in this disease (Souhami *et al.*, 1991). This is due to the fact that the majority of previous studies have employed immunohistochemical methods for demonstration of NCAM; such methods do not allow distinction between the various NCAM isoforms. In one study of NCAM expression in seven SCLC cell lines by immunoblotting (Aletsee-Ufrecht *et al.*, 1990), only the NCAM-135 isoform was demonstrated (termed NCAM 140 in their study). In two other previous studies in which SCLC NCI-H69 was included (Kibbelaar *et al.*, 1989; Moolenaar *et al.*, 1990), this tumour was shown to express both NCAM-190 and NCAM-135. The expression of these two major isoforms in NCI-H69 was confirmed in the present study. We found coexpression of NCAM-190 and NCAM-135 in more than half of the examined tumours, demonstrating that, in SCLC, two NCAM isoforms are commonly co-expressed. Since the role of the different NCAM isoforms is not settled, the possible biological implications of the difference in NCAM isoform expression between various SCLC tumours remains obscure.

It has recently been demonstrated, that the antigen recognised by SCLC cluster one (Cl 1) antibodies (Souhami *et al.*, 1991) is identical to NCAM (Patel *et al.*, 1989; Moolenaar *et al.*, 1990). Most SCLC tumours investigated so far have been found to express NCAM, while expression is found in only a few non-SCLC lung tumours (Mooi *et al.*, 1988; Kibbelaar *et al.*, 1989; Aletsee-Ufrecht *et al.*, 1990).

We detected polysialylation in 14/18 tumours (Table I), but we cannot rule out the possibility that a lower level of polysialylation may be present in the tumours scored as negative. Low levels of polysialylation may be detectable with antibodies with high affinity for α2,8-linked polysialic acid side chains. Polysialylation of NCAM profoundly reduces the homophilic binding properties of cells (Rutishauser *et al.*, 1988) and may facilitate migration of the cells due to altered cell-cell interactions. Thus, it could be speculated that the presence of polysialylation of NCAM on SCLC cells may contribute to the highly metastatic behaviour of this tumour type.

Qualitatively, the expression pattern of cadherin and NCAM was independent of whether the individual tumours were grown *in vitro* as cell lines or *in vivo* as nude mouse xenografts. However, a tendency towards higher expression in cell lines than in xenografts was noted. The difference may to some extent be caused by the presence in xenografts of murine stroma, which causes a dilution of the tumour cells. Despite the possible slight difference in the level of cadherin and NCAM expression between cell lines and xenografts, the demonstration of these adhesion molecules both in cell lines and in xenografts is evidence that expression is an inherent characteristics of the SCLC tumour cells, and not a phenotypic feature induced by the growth conditions. This notion is supported by the consistent demonstration of NCAM expression in SCLC in surgical biopsies (Mooi *et al.*, 1988; Kibbelaar *et al.*, 1989; Tome *et al.*, 1990), as well as in cell lines established from patients with this disease (Kibbelaar *et al.*, 1989; Moolenaar *et al.*, 1990; Aletsee-Ufrecht *et al.*, 1990).

It has been suggested that NCAM expression may be a feature specific of SCLC cell lines growing as floating aggregates in *in vitro* culture whereas cells growing adherent to the bottom of culture flasks express less or no NCAM (Doyle *et al.*, 1990). Our data do not support this hypothesis since the CPH cell lines, the DMS cell lines except DMS-79, and GLC-3 grew attached to the bottom of culture flasks. Thus, 10/14 cell lines expressed NCAM and grew attached.

At least three different types of cell-cell adhesion molecules are expressed on SCLC cells. Apart from cadherin and NCAM, also $\beta_1$ integrin is expressed in a large proportion of SCLC cells (Feldman *et al.*, 1991). Cell-cell adhesion molecules are likely to be involved in the process of metastasis (Nicolson, 1988; Thiery *et al.*, 1988; Takeichi, 1991). However, it is not clear what influence adhesion molecules on the surface of cancer cells have on their ability to metastasise. On one hand adhesion molecules may retard the escape of tumour cells from the primary site due to increased adhesion to other cells and to intercellular matrix proteins, but on the other hand adhesion molecules may be necessary for the cells to attach to a secondary, i.e. metastatic, site. However, decreased metastatic potential of cells expressing NCAM was reported (Andersson *et al.*, 1991) and also increased invasive and metastatic potential upon loss of cadherin expression (Behrens *et al.*, 1989; Hashimoto *et al.*, 1989; Vleminckx *et al.*, 1991) has been described. Hence, the functional role of adhesion molecules in invasion and metastasis is complex, and it may seem surprising that SCLC which is highly metastatic in patients, expresses several types of adhesion molecules.

The authors thank Klaus Edvardsen for providing the cadherin antibody. We gratefully acknowledge the financial support of the Danish Cancer Society, the Novo Foundation, the Lily Benthine Lund Foundation, the Michaelsen Foundation, King Christian X's Foundation, the Torben Linnemann Foundation, the Foundation of 17-12-81, P. Carl Petersens Foundation, the Astrid Thaysen Foundation, and the Foundation of 1870. The skillful technical assistance of Jette Røhrmann is greatly appreciated.

### References

ALETSEE-UFRECHT, M.C., LANGLEY, K., ROTSCH, M., HAVEMANN, K. & GRATZL, M. (1990). NCAM: a surface marker for human small cell lung cancer cells. *FEBS*, **267**, 295.

ANDERSSON, A.M., MORAN, N., GAARDSVOLL, H. & 4 others (1991). Characterization of NCAM expression and function in BT4C and $BT4C_n$ glioma cells. *Int. J. Cancer*, **47**, 124.

BEHRENS, J., MAREEL, M.M., VAN ROY, F.M. & BIRCHMEIER, W. (1989). Dissecting tumor cell invasion: epithelial cells acquire invasive properties after the loss of uvomorulin-mediated cell-cell adhesion. *J. Cell Biol.*, **108**, 2435.

BERENDSEN, H.H., DE LEIJ, L., DE VRIES, E.G.E. & 8 others (1988). Characterization of three small cell lung cancer cell lines established from one patient during longitudinal follow-up. *Cancer Res.*, **48**, 6891.

BOCK, E. (1991). Cell-cell adhesion molecules. *Biochem. Soc. Trans.* In the press.

BRADFORD, M.M. (1976). A rapid and sensitive method for the quantitation of microgram quantities of protein utilizing the principle of protein-dye binding. *Anal. Biochem.*, **72**, 248.

CARNEY, D.N., GAZDAR, A.F, BEPLER, G. & 5 others (1985). Establishment and identification of small cell lung cancer cell lines having classic and variant features. *Cancer Res.*, **45**, 2913.

DE LEIJ, L., POSTMUS, P.E., BUYS, C.H.C.M. & 7 others (1985). Characterization of three new variant type cell lines derived from small cell carcinoma of the lung. *Cancer Res.*, **45**, 6024.

DOYLE, L.A., BORGES, M., HUSSAIN, A., ELIAS, E. & TOMIYASU, T. (1990). An adherent subline of a unique small-cell lung cancer cell line downregulates antigens of the neural cell adhesion molecule. *J. Clin. Invest.*, **86**, 1848.

ENGELHOLM, S.A., VINDELØV, L.L., SPANG-THOMSEN, M. & 4 others (1985). Genetic instability of cell lines derived from a single human small cell carcinoma of the lung. *Eur. J. Cancer Clin. Oncol.*, **21**, 815.

ENGELHOLM, S.A., SPANG-THOMSEN, M., VINDELØV, L.L. & 5 others (1986). Comparison of characteristics of human small cell carcinoma of the lung in patients, *in vitro* and transplanted into nuce mice. *Acta Pathol. Microbiol. Immunol. Scand. Sect. A*, **94**, 325.

FELDMAN, L.E., SHIN, K.C., NATALE, R.B. & TODD, R.F. (1991). $\beta_1$ integrin expression on human small cell lung cancer cells. *Cancer Res.*, **51**, 1065.

FINNE, J. (1982). Occurrence of unique polysialosyl carbohydrate units in glycoproteins of developing brain. *J. Biol. Chem.*, **257**, 11966.

GEIGER, B., VOLBERG, T., GINSBERG, D., BITZUR, S., SABANAY, I. & HYNES, R.O. (1990). Broad spectrum pan-cadherin antibodies, reactive with the C-terminal 24 amino acid residues of N-cadherin. *J. Cell Sci.*, **97**, 607.

HASHIMOTO, M., NIWA, O., NITTA, Y., TAKEICHI, M. & YOKORO, K. (1989). Unstable expression of E-cadherin adhesion molecules in metastatic ovarian tumour cells. *Jpn. J. Cancer Res.*, **80**, 459.

HOFFMAN, S. & EDELMAN, G.M. (1983). Kinetics of homophilic binding by embryonic and adult forms of the neural cell adhesion molecule. *Proc. Natl Acad. Sci. USA*, **80**, 5762.

KIBBELAAR, R.E., MOOLENAAR, C.E.C., MICHALIDES, R.J.A.M., BITTER-SUERMANN, D., ADDIS, B.J. & MOOI, W.J. (1989). Expression of the embryonal neural cell adhesion molecule N-CAM in lung carcinoma. Diagnostic usefulness of monoclonal antibody 735 for the distinction between small cell lung cancer and non-small cell lung cancer. *J. Pathol.*, **159**, 23.

KYHSE-ANDERSEN, J. (1984). Electroblotting of multiple gels: a simple apparatus without buffer tank for rapid transfer of proteins from polyacrylamide to nitrocellulose. *J. Biochem. Biophys. Methods*, **10**, 203.

LAEMMLI, U.K. (1970). Cleavage of structural proteins during the assembly of the head of bacteriophage T4. *Nature*, **227**, 680.

LAGUNOWICH, L.A, DONOSO, L.A. & GRUNWALD, G.B. (1990). Identification of mammalian and invertebrate analogues of the avian calcium-dependent cell adhesion protein N-cadherin with synthetic-peptide directed antibodies against a conserved cytoplasmic domain. *Biochem. Biophys. Res. Commun.*, **172**, 313.

LINNEMANN, D. & BOCK, E. (1989). Cell adhesion molecules in neural development. *Dev. Neurosci.*, **11**, 149.

LINNEMANN, D., RAZ, A. & BOCK, E. (1989). Differential expression of cell adhesion molecules in variants of K1735 melanoma cells differing in metastatic capacity. *Int. J. Cancer*, **43**, 709.

MOOI, W.J., WAGENAAR, S.S., SCHOL, D. & HILGERS, J. (1988). Monoclonal antibody 123C3 in lung tumour classification. Immunohistology of 358 resected lung tumours. *Mol. Cell. Probes*, **2**, 31.

MOOLENAAR, C.E.C.K., MULLER, E.J., SCHOL, D.J. & 4 others (1990). Expression of neural cell adhesion molecule-related sialoglycoprotein in small cell lung cancer and neuroblastoma cell lines H69 and CHP-212. *Cancer Res.*, **50**, 1102.

NICOLSON, G.L. (1988). Organ specificity of tumor metastasis: role of preferential adhesion, invasion and growth of malignant cells at specific secondary sites. *Cancer Metast. Rev.*, **7**, 145.

NYBROE, O., LINNEMANN, D. & BOCK, E. (1988). NCAM biosynthesis in brain. *Neurochem. Int.*, **12**, 251.

OWENS, G.C., EDELMAN, G.M. & CUNNINGHAM, B.A. (1987). Organization of the neural cell adhesion molecule (N-CAM) gene: alternative exon usage as the basis for different membrane-associated domains. *Proc. Natl Acad. Sci. USA*, **84**, 294.

PATEL, K., MOORE, S.E., DICKSON, G. & 4 others (1989). Neural cell adhesion molecule (NCAM) is the antigen recognized by monoclonal antibodies of similar specificity in small-cell lung carcinoma and neuroblastoma. *Int. J. Cancer*, **44**, 573.

PETTENGILL, O.S., SORENSON, G.D., WURSTER-HILL, D. & 4 others (1980). Isolation and growth characteristics of continuous cell lines from small-cell carcinoma of the lung. *Cancer*, **45**, 906.

RASMUSSEN, S., BEREZIN, V., NØRGAARD-PEDERSEN, B. & BOCK, E. (1983). Purification of the glycoprotein D2 from fetal and adult human brain. In *Protides of the Biological Fluids*, XXX Colloquium, Peeters, H. (ed.), p. 83, Pergamon Press: Oxford, UK.

RUTISHAUSER, U., ACHESON, A., HALL, A.K., MANN, D.M. & SUNSHINE, J. (1988). The neural cell adhesion molecule (NCAM) as a regulator of cell-cell interactions. *Science*, **240**, 53.

SANTONI, M.J., BARTHELS, D., VOPPER, G., BONED, A., GORIDIS, C. & WILLE, W. (1989). Differential exon usage involving an unusual splicing mechanism generates at least eight types of NCAM cDNA in mouse brain. *EMBO J.*, **8**, 385.

SCHOL, D.J., MOOI, W.J., VAN DER GUGTEN, A.A., WAGENAAR, S.S. & HILGERS, J. (1988). Monoclonal antibody 123C3, identifying small cell carcinoma phenotype in lung tumours, recognizes mainly, but not exclusively, endocrine and neuron-supporting normal tissues. *Int. J. Cancer*, **2** (Suppl), S34.

SHIMOYAMA, Y., HIROHASHI, S., HIRANO, S. & 4 others (1989). Cadherin cell-adhesion molecules in human epithelial tissues and carcinomas. *Cancer Res.*, **49**, 2128.

SOUHAMI, R.L., BEVERLEY, P.C.L., BOBROW, L.G. & LEDERMANN, J.A. (1991). Antigens of lung cancer: results of the second international workshop on lung cancer antigens. *J. Natl Cancer Inst.*, **83**, 609.

SPANG-THOMSEN, M., NIELSEN, A. & VISFELDT, J. (1980). Growth curves of three human malignant tumors transplanted to nude mice. *Exp. Cell Biol.*, **48**, 138.

SUZUKI, S., SANO, K. & TANIHARA, H. (1991). Diversity of the cadherin family: evidence for eight new cadherins in nervous tissue. *Cell Regul.*, **2**, 261.

TAKEICHI, M. (1991). Cadherin cell adhesion receptors as a morphogenic regulator. *Science*, **251**, 1451.

THIERY, J.-P., BOYER, B., TUCKER, G., GAVRILOVIC, J. & VALLES, A.-M. (1988). Adhesion mechanisms in embryogenesis and in cancer invasion and metastasis. *CIBA Found. Symp.*, **141**, 48.

TOME, Y., HIROHASHI, S., NOGUCHI, M. & SHIMOSATO, Y. (1990). Preservation of cluster 1 small cell lung cancer antigen in zinc-formalin fixative and its application to immunohistological diagnosis. *Histopathology*, **16**, 469.

VLEMINCKX, K., VAKAET, L., MAREEL, M., FIERS, W. & VAN ROY, F. (1991). Genetic manipulation of E-cadherin expression by epithelial tumor cells reveals an invasion suppressor role. *Cell*, **66**, 107.